# Structure-based identification of salvianolic acid B as an inhibitor targeting *Salmonella* InvC ATPase

**DOI:** 10.1016/j.jbc.2025.110722

**Published:** 2025-09-15

**Authors:** Jiayang Liu, Xinyou Zhang, Kaiyao Zhang, Jianfeng Wang, Xuming Deng, Hongtao Liu, Yanhong Deng, Jiazhang Qiu

**Affiliations:** State Key Laboratory for Diagnosis and Treatment of Severe Zoonotic Infectious Diseases, Key Laboratory for Zoonosis Research of the Ministry of Education, College of Veterinary Medicine, Jilin University, Changchun, China

**Keywords:** *Salmonella*, type III secretion system (T3SS), ATPase, natural product, virulence factor, molecular docking

## Abstract

Multidrug-resistant (MDR) *Salmonella* poses a significant global health threat. The Type III Secretion System 1 (T3SS-1) and its ATPase InvC are crucial for virulence and promising drug targets. Through structure-based virtual screening, we identified Salvianolic acid B (SA-B) as an inhibitor of InvC. To rigorously characterize its interaction, we performed extensive molecular dynamics simulations, which revealed a 'dynamic yet stable' binding mode within the ATP-binding pocket. Subsequent experimental validation confirmed that SA-B directly binds the InvC ATP pocket and inhibits its ATPase activity. Consequently, SA-B inhibited T3SS-1-mediated effector secretion and reduced the invasion of host cells by *S*. Typhimurium *in vitro*, without affecting bacterial viability. Importantly, SA-B demonstrated significant therapeutic efficacy in *Galleria mellonella* and mouse infection models, improving survival and reducing bacterial burden. This study establishes SA-B as a promising anti-virulence lead compound targeting *Salmonella* InvC, offering a strategy that may mitigate antibiotic resistance by selectively disarming pathogen virulence mechanisms rather than targeting viability.

Despite decades of medical advances, *Salmonella* infections remain an alarming threat to global public health. These infections primarily manifest as gastroenteritis and enteric fever. Gastroenteritis caused by *Salmonella* is estimated to result in approximately 93.8 million cases annually, causing approximately 155,000 deaths (1). Of particular concern is the substantial burden imposed by typhoid and paratyphoid fever, which together account for approximately 15.5 million cases and 154,000 deaths each year (2, 3). Among various *Salmonella* strains, *S.* typhimurium has emerged as a paradigmatic model for investigating mechanisms of infection, owing to its prevalent association with gastroenteritis in both human and animal hosts. The increasing prevalence of multidrug-resistant *Salmonella* strains, including *S*. typhimurium variants, which exhibit resistance to commonly used antibiotics such as ampicillin, chloramphenicol, tetracyclines, and trimethoprim-sulfamethoxazole, presents an alarming development (4). This resistance significantly compromises the effectiveness of conventional therapies, posing a formidable challenge for managing *Salmonella* infections (5, 6, 7). Facing this escalating resistance crisis, researchers are actively exploring alternative drug targets and innovative strategies designed to overcome the inherent limitations of traditional antibiotics (8, 9).

One promising strategy to counteract this resistance crisis is the development of anti-virulence drugs. By neutralizing virulence factors, these agents can limit infection-induced tissue damage and inflammation, thereby reducing disease severity and allowing more time for the host immune system and conventional antibiotics to clear the pathogen (10, 11, 12). This approach avoids the dangerous paradoxical effects seen with some antibiotics, which can inadvertently increase toxin expression, and thus offers safer adjunctive therapy (10). Unlike traditional antibiotics that directly target bacterial growth or survival and thus exert strong selective pressure for resistance, anti-virulence therapies aim to disarm pathogens by inhibiting virulence factors essential for infection but not for basic viability (13, 14), thereby slowing the development of resistance. Current anti-virulence strategies being used or under development target a variety of biological components in pathogenic bacteria, including adhesins, toxins, secretion systems, quorum sensing molecules, and biofilms (14, 15). The Type III Secretion System 1 (T3SS-1), a complex molecular machine composed of multiple interacting components, is a particularly attractive target for anti-virulence strategies due to its pivotal role in bacterial pathogenesis through the injection of effector proteins into host cells (14, 16). Among these, InvC, the ATPase encoded by *Salmonella* Pathogenicity Island 1 (SPI-1), plays an indispensable role (17). As an ATP-driven motor, InvC provides the energy required for effector protein translocation and is crucial for orchestrating the entire secretion process, including substrate protein unfolding (17, 18). Its essential function in *Salmonella* virulence makes InvC a highly promising therapeutic target. In contrast to the reported inhibitors targeting T3SS ATPases from various pathogens, including *Yersinia* (19), *Burkholderia mallei* (19), *Shigella* (20), and *Chlamydia* (21), limited research has been conducted on inhibitors specifically targeting *Salmonella* InvC. Consequently, there is a pressing need to identify effective InvC inhibitors.

To address this need, we turned to natural products, which have historically served as invaluable sources of drug leads, especially in the field of anti-infective therapy, owing to their diverse and complex chemical structures (22, 23). Salvianolic acid B (SA-B), a major water-soluble component of *Salvia miltiorrhiza*, is known for a wide spectrum of pharmacological activities, including antioxidant, anti-inflammatory, and anti-cancer properties (24, 25). Growing evidence from recent studies investigating the effects of SA-B against pathogenic bacteria like *Neisseria meningitidis* and *Acinetobacter baumannii* indicates its broad-spectrum anti-infective potential (26, 27). However, the specific mechanisms underlying the anti-infective activity of SA-B, particularly its potential in targeting bacterial virulence, have not been thoroughly explored.

In this study, we employed a structure-based virtual screening strategy and identified SA-B as a potential inhibitor of *Salmonella* InvC. We validated its inhibitory activity on InvC ATPase through biochemical assays and confirmed its anti-virulence effect in *S*. Typhimurium without affecting bacterial viability. Furthermore, *in vivo* infection models demonstrated its therapeutic efficacy. These findings not only elucidate a specific anti-virulence function of SA-B but also provide a rational basis for developing anti-virulence therapies targeting *Salmonella* T3SS.

## Results

### Discovery of SA-B as a small-molecule inhibitor of InvC through docking analysis

**Figure 1:**
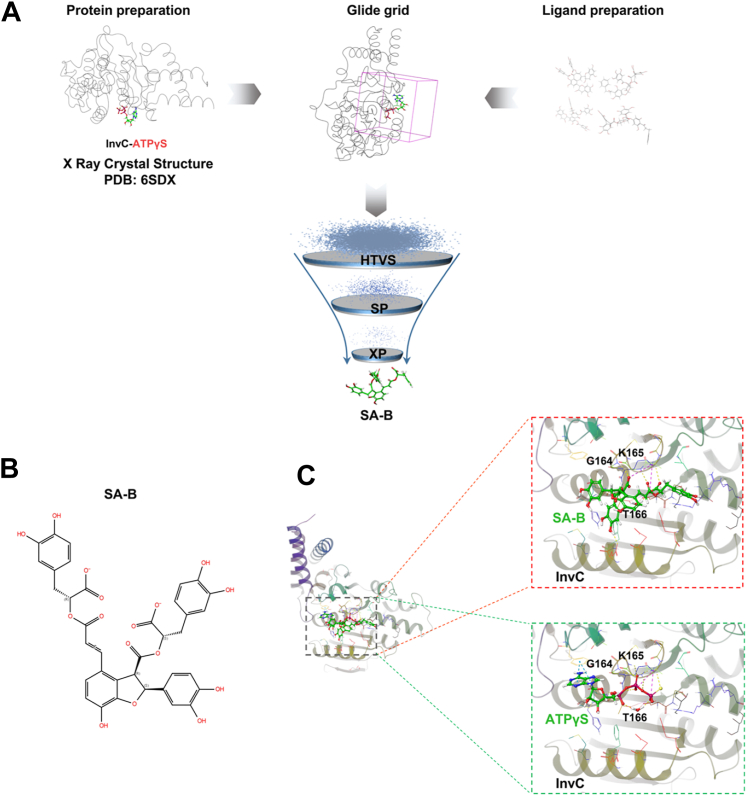
**Virtual screening identifies SA-B as a potential InvC inhibitor.***A*, schematic of the structure-based virtual screening workflow. The crystal structure of the InvC-ATPγS complex (PDB ID: 6SDX) was used as the receptor. A natural product library was screened using a tiered approach of HTVS, SP, and XP docking, which identified SA-B as the top-ranking hit. *B*, the chemical structure of SA-B. *C*, comparison of the predicted binding pose of SA-B and the co-crystallized ligand ATPγS within the active site of InvC. The insets highlight interactions with the key P-loop residues G164, K165, and T166.

To identify small-molecule inhibitors targeting the ATPase InvC, a structure-based virtual screening approach was employed, leveraging the crystal structure of the InvC-ATPγS complex (PDB ID: 6SDX) (17), which both bypassed the limitations and potential inaccuracies associated with homology modeling and, with its co-crystallized ATPγS, precisely defined the true ligand-binding site, thereby ensuring highly reliable and accurate virtual screening. The stereochemical quality of the 6SDX structure was rigorously re-evaluated using MolProbity (Fig. S1). Analysis revealed that 94.2% (327/347) of all residues were located in favored (98%) regions of the Ramachandran plot, and 100.0% (347/347) were within allowed (>99.8%) regions, with no outliers. This assessment confirmed the exceptionally high stereochemical quality of the protein backbone conformation. The multi-tiered screening workflow involved a sequential docking strategy integrating high-throughput virtual screening (HTVS), standard precision (SP), and extra precision (XP) docking (28). These computational techniques, widely used in computer-aided drug design for identifying potential anti-infective agents (29, 30, 31, 32), facilitated the systematic evaluation of candidate ligands based on predicted binding affinities, enabling the identification of promising inhibitors (Fig. 1*A*). Before commencing the formal screening, the reliability and reproducibility of the docking procedure were validated (29). This validation step involved XP redocking the native ligand, ATPγS, into the active site of InvC. To assess the stability of the poses, both the redocked conformation and the native conformation (from the crystal structure) were subjected to independent 200 ns MD simulations. The average ligand root mean square deviation (RMSD) values for the redocked and native conformations over 200 ns were found to be 2.65 Å and 2.78 Å (Fig. S2), respectively. These results confirm that the redocking protocol is capable of reproducing a stable pose comparable to the native conformation, thereby supporting subsequent screening steps.

From a library of 2609 natural product candidates (Supporting Information S1), SA-B was identified as the top-ranking hit with a docking score of −14.336 kcal/mol (Fig. 1*B*). Molecular docking analysis revealed that SA-B occupies the ATP-binding pocket of InvC, adopting a pose that closely mirrors the co-crystallized ligand ATPγS. Both SA-B and ATPγS form multiple contacts with residues G164, K165, and T166 in the P-loop, a motif critical for ATP binding (17, 33), which highlights a shared binding mechanism (Fig. 1*C*). These findings indicate that SA-B could occupies the ATP-binding site, potentially disrupting the ATPase activity essential for InvC function.

### Insights into SA-B-InvC interactions through MD simulation

**Figure 2:**
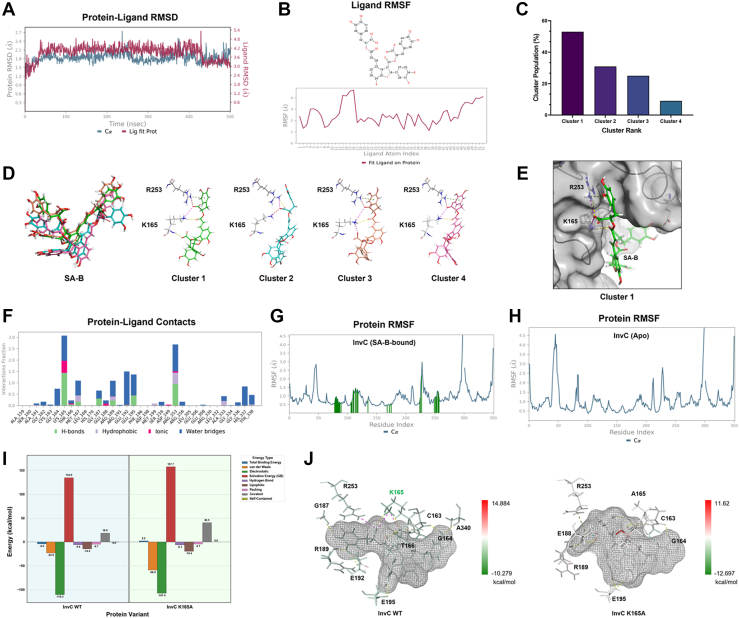
**MD simulation and energetic calculations characterize the SA-B and InvC interaction.***A*, RMSD of the InvC protein backbone (Cα, *blue*) and the SA-B ligand (*red*) over a 500 ns MD simulation. *B*, RMSF of the SA-B ligand atoms. The top panel shows the 2D structure of SA-B, and the bottom panel plots the RMSF value for each atom. *C*, population distribution of the four major conformational clusters of SA-B identified during the simulation. *D*, superimposition of the four dominant conformational clusters. Interaction diagrams for the representative pose of each cluster highlight contacts with key residues K165 and R253. *E*, a detailed view of the most populated conformation, cluster 1, within the InvC binding pocket. *F*, a statistical summary of the interactions, including hydrogen bonds, hydrophobic interactions, ionic bonds, and water bridges, between SA-B and InvC. *G* and *H*, comparison of the protein RMSF profiles for InvC in its SA-B-bound state (*G*) and unbound (Apo) state (*H*). *I*, MM-GBSA binding free energy decomposition for SA-B in complex with wild-type (WT) InvC and the K165A mutant. *J*, Per-residue energy contribution map for the interaction of SA-B with WT InvC and the K165A mutant. The ligand, SA-B, is shown as a mesh surface. Residues within the binding pocket are colored by energy contribution, where *green* indicates favorable interactions and *red* indicates unfavorable interactions.

Since molecular docking provides only a static snapshot, we performed a 500 ns molecular dynamics (MD) simulation to investigate the dynamic stability and interaction characteristics of the SA-B-InvC complex (29, 30, 31, 32). After the initial equilibration phase, the InvC protein backbone remained stable, with the RMSD fluctuating between 1 and 3 Å during the simulation (Fig. 2*A*, blue trace). In contrast, the RMSD of the SA-B ligand relative to the InvC protein remained within 3 to 5 Å (Fig. 2*A*, red trace). This observed ligand mobility is likely attributable to the large size and significant inherent flexibility of the SA-B molecule. This flexibility is confirmed by the ligand's root-mean-square fluctuation (RMSF) analysis (Fig. 2*B*), which shows pronounced movement in its side chains, particularly at atoms 13 to 16 and 47 to 52. To further characterize this dynamic binding mode, we performed a cluster analysis on the simulation trajectory, which identified four predominant conformational clusters of SA-B within the binding pocket (Fig. 2*C*). A superimposition of these four conformations (Fig. 2*D*) reveals that the regions with high RMSF values correspond to the flexible portions of the ligand. Cluster one represents the most frequently occurring, and thus most dominant, conformation (Fig. 2*E*). Most importantly, despite this flexibility, all major conformations maintain persistent interactions with residues K165 and R253 (Fig. 2*D*). The binding is primarily anchored by a robust network of hydrogen bonds, hydrophobic interactions, and water bridges, as detailed in the statistical analysis of interaction types (Fig. 2*F*). This analysis reveals a 'dynamic yet stable' binding mode, where the flexible side chains of SA-B adapt conformationally while its core structure remains firmly anchored within the InvC active site. To demonstrate the stabilizing effect of this interaction on the protein itself, a separate 500 ns simulation of unbound (Apo) InvC was performed. The comparison confirmed that SA-B binding reduces overall protein flexibility, with the average protein RMSF decreasing from 1.166 Å (Apo state) to 1.055 Å (SA-B-bound state) (Fig. 2, *G* and *H*).

The persistent interaction with K165, a key P-loop residue responsible for forming a salt bridge with the β-phosphate of ATP, identified it as a critical anchor point for SA-B binding. To quantify the pivotal role of K165, MM-GBSA calculations compared the binding energetics of WT InvC with an *in silico* K165A mutant. The mutation caused a stark loss of affinity, with the binding free energy (Δ*G* bind) shifting from −4.0 to +2.2 kcal/mol. This destabilization was driven primarily by significant penalties in covalent and solvation energies that outweighed compensatory van der Waals interactions (Fig. 2*I*). This conclusion is visually supported by per-residue energy decomposition (Fig. 2*J*), which shows that the mutation abolishes the major favorable binding contribution from the K165 residue. Notably, the mutation also nullified the favorable binding contributions from adjacent residues, including C163, T166, and A340. Collectively, these computational analyses support the hypothesis that SA-B acts as a competitive ATP-binding site inhibitor by occupying and adapting within the active site.

### SA-B disrupts T3SS-1 effector secretion through InvC ATPase inhibition

**Figure 3:**
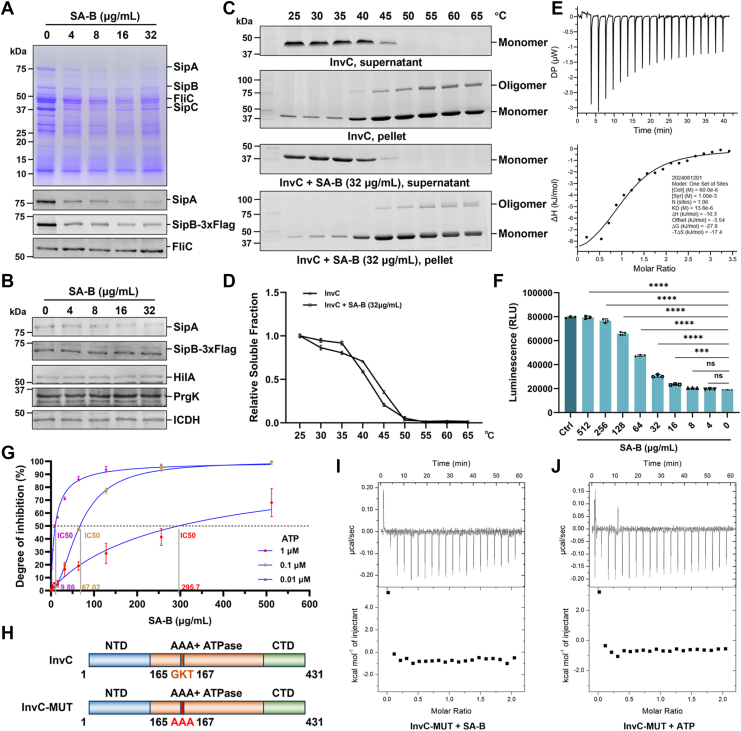
**SA-B inhibits T3SS-1 function by targeting the ATPase activity of InvC.***A*, SA-B inhibits the secretion of T3SS-1 effector proteins. *S. Typhimurium* was cultured under T3SS-1-inducing conditions with indicated concentrations of SA-B. Secreted proteins in the supernatant were analyzed by Coomassie blue staining (*top*) and western blotting for SipA and SipB-3xFlag (*bottom*). FliC serves as a negative control. *B*, analysis of intracellular levels of T3SS-1-related proteins. Bacterial lysates were analyzed by western blotting for SipA, SipB-3xFlag, HilA, and PrgK. ICDH was used as a loading control. *C* and *D*, TSA of InvC thermal stability. Purified InvC (5 μg) was incubated with or without SA-B (32 μg/ml), heated at the indicated temperatures for 5 min, and separated into soluble (supernatant) and insoluble (pellet) fractions by centrifugation. Fractions were analyzed by SDS-PAGE (*C*), and the relative soluble fraction was quantified (*D*). *E*, ITC analysis of SA-B binding to InvC. *F*, measurement of InvC ATPase activity. The enzymatic activity of purified InvC (1 μM) was measured in the presence of increasing concentrations of SA-B at a fixed ATP concentration of 0.1 μM, using an ATP-based luminescence assay. The control (Ctrl) reaction lacked the InvC protein. *G*, determination of IC_50_ values for SA-B against InvC. The assay was performed at varying ATP concentrations of 0.01, 0.1, and 1 μM. *H*, schematic representation of the wild-type (WT) InvC and the P-loop mutant (InvC-MUT) carrying G164A, K165A, and T166 A substitutions. *I* and *J*, ITC analysis of ligand binding to InvC-MUT. The binding of SA-B (*I*) and ATP (*J*) to the mutant protein was assessed. For panels with error bars (*D*, *F*, and *G*), data are shown as mean ± SD from three independent experiments. For panels *A*–*C*, *E*, *I*, and *J*, data are representative of three independent experiments. Statistical significance for panel F was determined using one-way ANOVA with Tukey's *post hoc* test. ∗∗∗*p* < 0.0001; ns, not significant.

To experimentally validate our computational hypothesis, we first investigated the effect of SA-B on T3SS-1 function. Biochemical and functional assays revealed that SA-B potently inhibited the secretion of key T3SS-1 effectors SipA, SipB, and SipC in a dose-dependent manner (Fig. 3*A*) (34). This effect was observed at concentrations as low as 4 μg/ml. In contrast, intracellular levels of T3SS-1-associated components, including SipA, SipB, HilA, and PrgK, remained unchanged under identical experimental conditions (Fig. 3*B*), confirming that SA-B specifically impacts the secretion process rather than protein expression or stability. Next, to confirm that this inhibition resulted from a direct interaction with InvC, the investigation began by using a thermal shift assay (TSA) as an initial screen for binding. Although SA-B did not cause a significant change in the thermal stability of InvC, we observed a distinct alteration in the oligomeric state of the denatured protein precipitate, hinting at a possible interaction (Fig. 3, *C* and *D*). To quantify this interaction more definitively, isothermal titration calorimetry (ITC) confirmed direct binding, yielding a dissociation constant (*K*_D_) of 13.8 μM (Fig. 3*E*). The thermodynamic signature revealed the interaction was enthalpically and entropically favorable, with Δ*H* = −10.3 kJ/mol and -*T*Δ*S* = −17.4 kJ/mol, resulting in a Gibbs free energy (Δ*G*) of −27.8 kJ/mol and indicating a specific, moderate-affinity interaction. The functional consequence of this interaction was subsequently assessed. An ATP-based luminescence assay revealed that SA-B inhibited the enzymatic activity of purified InvC in a dose-dependent manner (Fig. 3*F*). At ATP concentrations of 0.01, 0.1, and 1 μM, the corresponding IC_50_ values for SA-B were 9.86, 67.02, and 295.7 μg/ml, respectively, demonstrating a characteristic feature of competitive inhibition (Fig. 3*G*). Finally, to further validate the binding site, a triple mutant of InvC designated InvC-MUT, containing G164A, K165A, and T166A substitutions in the P-loop, was constructed (Fig. 3*H*). Consistent with a shared binding site, subsequent ITC analysis confirmed that the mutant protein was unable to bind to either SA-B or ATP (Fig. 3, *I* and *J*). Taken together, these results demonstrate that SA-B competitively binds to the ATP-binding pocket of InvC, inhibiting its ATPase activity and thereby disrupting T3SS-1 effector secretion.

### SA-B specifically inhibits *S.* typhimurium invasion without affecting adhesion and replication

**Figure 4:**
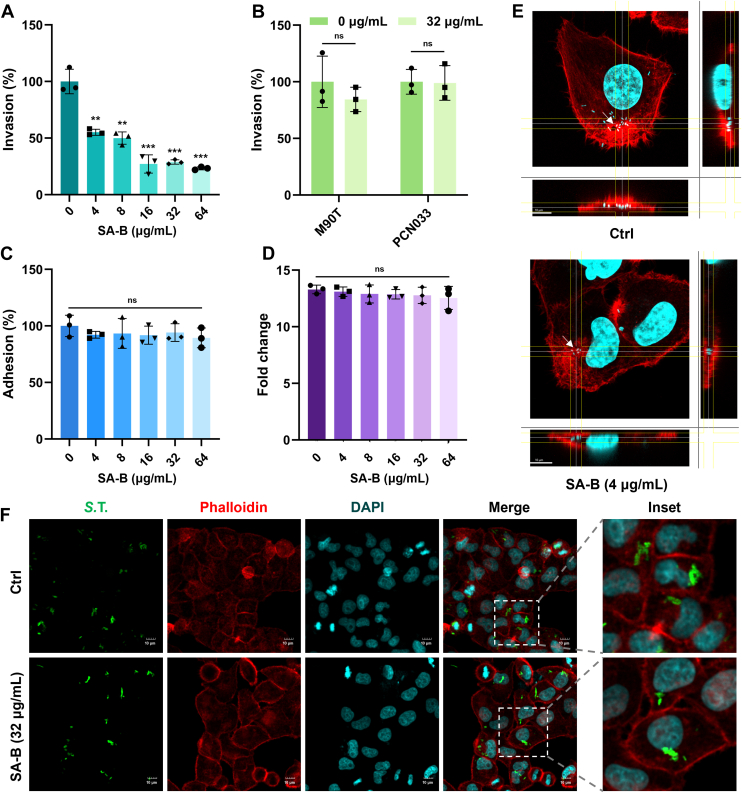
**SA-B selectively inhibits *S.* Typhimurium invasion of HeLa cells.***A*, invasion assay of *S.* Typhimurium in HeLa cells. Bacteria were pre-treated with SA-B at the indicated concentrations for 4 h and then used to infect HeLa cells for 1 h at an MOI of 100. *B*, invasion assay of *S. flexneri* M90 T and *E. coli* PCN033 in HeLa cells. Bacteria were pre-treated with or without SA-B (32 μg/ml) for 4 h. HeLa cells were then infected for 1 h at an MOI of 50 for *S. flexneri* and 100 for *E. coli*. *C*, adhesion assay of *S.* Typhimurium to HeLa cells. Bacteria were pre-treated with SA-B at the indicated concentrations for 4 h and then used to infect HeLa cells for 1 h at an MOI of 100. Adherent bacteria were subsequently quantified. *D*, intracellular replication assay of *S.* Typhimurium in HeLa cells. Cells were infected for 1 h at an MOI of 20. After killing extracellular bacteria, the cells were incubated for 24 h in medium containing SA-B at the indicated concentrations. Intracellular bacterial replication was then quantified. *E*, representative confocal microscopy Z-stack images confirming intracellular localization of *S.* Typhimurium during invasion. The top images represent the control group, and the bottom images represent the group treated with 4 μg/ml SA-B. Orthogonal views show bacteria, indicated by *white arrows*, located inside the host cells. F-actin is stained *red* by Phalloidin, while nuclei and bacteria are stained cyan by DAPI. *F*, representative immunofluorescence images from the intracellular replication assay of HeLa cells infected with *S. Typhimurium* and treated with or without 32 μg/ml SA-B. Bacteria appear *green*, F-actin is stained *red* with Phalloidin, and nuclei are stained cyan with DAPI. Scale bar, 10 μm. For *panels* with error bars (*A*, *C*, and *D*), data are presented as mean ± SD from three independent experiments. Statistical significance was determined using one-way ANOVA with Tukey's *post hoc* test for *panelsA*, *C*, and *D*, and an unpaired, two-tailed Student's *t* test for *panelB*. ∗∗*p* < 0.05; ∗∗∗*p* < 0.001; ns, not significant.

Given that T3SS-1-mediated effector protein secretion is essential for host cell entry, we next hypothesized that the observed inhibition of secretion by SA-B would translate into a reduced capacity for *S.* Typhimurium to invade host cells. As expected, SA-B inhibited *S.* Typhimurium invasion in a dose-dependent manner, achieving nearly 50% inhibition at a concentration as low as 4 μg/ml (Fig. 4*A*), and exhibited specificity for *S.* Typhimurium, as it had no effect on the invasion of *Shigella flexneri* or *Escherichia coli* (Fig. 4*B*). To confirm that this anti-invasion activity was not an indirect consequence of other effects, several control experiments were performed. Crucially, SA-B did not significantly impact bacterial adhesion to host cells or intracellular replication under the tested conditions (Fig. 4, *C* and *D*). These findings on bacterial invasion and replication were further confirmed by immunofluorescence analysis, which provided visual evidence consistent with the CFU assays (Fig. 4, *E* and *F*). Furthermore, SA-B showed no direct antibacterial activity, with a minimum inhibitory concentration (MIC) exceeding 1024 μg/ml, and it was non-toxic to HeLa cells at concentrations up to 128 μg/ml (Figs. S3, *A* and *B* and S4). This initial characterization identifies SA-B as a non-toxic anti-virulence agent that selectively disrupts the *Salmonella* invasion process.

### SA-B demonstrates therapeutic efficacy in *G. mellonella* and mouse models of *S.* typhimurium infection

**Figure 5:**
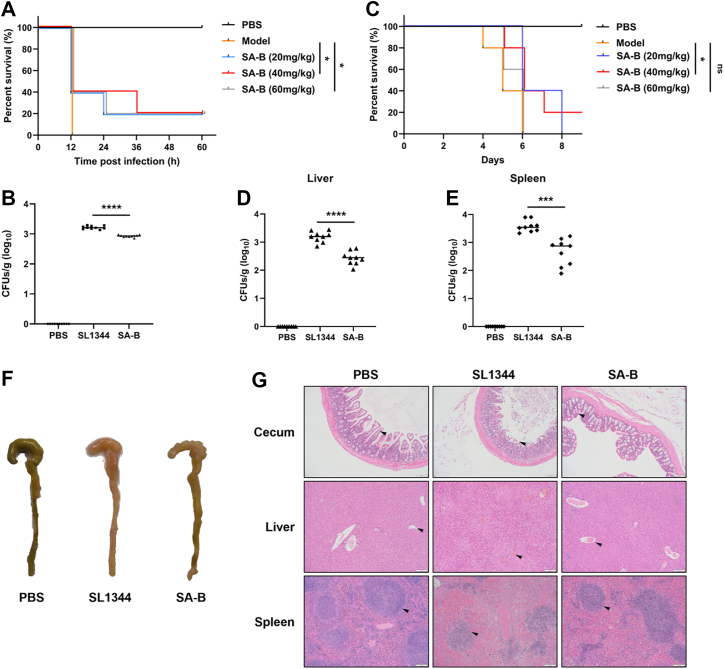
**Therapeutic efficacy of SA-B in *G. mellonella* and mouse models of *S.* Typhimurium infection.***A*, survival curves of *G. mellonella*. Groups of 10 larvae were infected with 1 × 10^5^ CFU of *S.* Typhimurium and subsequently treated with SA-B at the indicated doses. Survival was monitored for 60 h. *B*, bacterial load in *G. mellonella*. Groups of 10 larvae were infected with 5 × 10^4^ CFU of *S.* Typhimurium and treated with SA-B at a dose of 40 mg/kg. *C*, survival curves of C57BL/6 mice. Groups of 10 streptomycin-pretreated mice were orally challenged with 1 × 10^7^ CFU of *S.* Typhimurium and treated with SA-B at the indicated doses. Survival was recorded for 8 days. *D* and *E*, bacterial loads in the liver and spleen, respectively, of infected mice. Groups of 10 mice were challenged with 5 × 10^6^ CFU of *S.* Typhimurium and treated with SA-B at a dose of 40 mg/kg. *F*, representative images of ceca from different treatment groups, showing gross pathological changes. *G*, representative H&E-stained sections of the cecum, liver, and spleen. Black triangles indicate key pathological changes. Scale bar, 200 μm. For survival curves in (*A* and *C*), data were analyzed using the log-rank test. Bacterial load data in (*B*, *D*, and *E*) are presented as mean ± SD and were analyzed using a one-way ANOVA with Tukey's *post hoc* test. Images in (*F* and *G*) are representative of each group. ∗*p* < 0.05; ∗∗∗∗*p* < 0.0001; ns, not significant.

To determine if the *in vitro* anti-virulence activity of SA-B translated to therapeutic efficacy, we evaluated the compound using a tiered *in vivo* testing strategy, first employing the *Galleria mellonella* model for preliminary assessment, followed by a more clinically relevant mouse model for validation. In the *G. mellonella* model, SA-B treatment conferred significant protection. It increased the survival rate at all tested concentrations, with 40 mg/kg identified as the optimal dosage (Fig. 5*A*). Consistent with this, treatment at this dose also significantly inhibited the proliferation of *S.* typhimurium within the larvae (Fig. 5*B*). Building on these promising findings, we next assessed the efficacy of SA-B in a mouse model of salmonellosis. Treatment with SA-B at 40 mg/kg significantly improved mouse survival over an 8-day period (Fig. 5*C*). This survival benefit was accompanied by a marked reduction in bacterial burdens in systemic organs, decreasing the CFU count by approximately one log in the liver and 0.5 logs in the spleen compared to the SL1344-infected group (Fig. 5, *D* and *E*). The protective effect of SA-B was also evident at the histological level. SA-B treatment substantially ameliorated infection-induced tissue damage, mitigating the severe intestinal villus detachment, hepatic vascular congestion, and splenic lymphoid follicle disruption observed in untreated mice (Fig. 5, *F* and *G*). These results affirm the *in vivo* efficacy of SA-B, demonstrating that its ability to inhibit the T3SS-1 translates into a tangible therapeutic benefit in live infection models.

## Discussion

The escalating crisis of antimicrobial resistance demands innovative therapeutic approaches (35). Anti-virulence therapies offer a promising strategy, as they aim to disarm pathogens by targeting key virulence factors, such as the T3SS, thereby imposing less selective pressure for the development of resistance (36, 37, 38). In this study, we identified and validated SA-B, a natural product, as an inhibitor of the *Salmonella* T3SS-1 ATPase InvC. Our findings demonstrate that SA-B effectively suppresses *Salmonella* virulence *in vitro* and *in vivo*, establishing it as a promising lead compound for developing anti-infectives.

Our biochemical assays revealed that SA-B acts as a competitive inhibitor of InvC, with its inhibitory activity being dependent on the substrate concentration. Under low ATP conditions of 0.01 μM, SA-B demonstrated a potent inhibitory effect with an IC_50_ of 9.86 μg/ml, equivalent to 13.7 μM. This level of potency positions SA-B favorably within the existing landscape of T3SS-targeting compounds. Specifically, its activity is consistent with the 7.4 μM to 70 μM range reported for other small molecules that directly target T3SS ATPases in pathogens such as *Yersinia*, *Chlamydia*, and *EPEC* (21, 39, 40). Furthermore, its potency is also comparable to that of other broader T3SS inhibitors, like fusaric acid, which inhibits the *Salmonella* T3SS with an IC_50_ of 53.5 μM (41, 42). Our findings also contribute to the growing body of literature on the multifaceted anti-infective properties of SA-B. While we have demonstrated the action of SA-B on InvC in *Salmonella*, previous studies have reported activity against *N. meningitidis* and *A. baumannii* through different mechanisms (26, 27), suggesting that SA-B may function as a multi-target agent depending on the pathogen. Our work clarifies the anti-virulence mechanism of SA-B specifically in the context of *Salmonella* infection.

The significant therapeutic efficacy observed in both *G. mellonella* and mouse models underscores the clinical potential of SA-B. However, interpreting the *in vivo* effects of the compound requires careful consideration of the known pleiotropic activities of SA-B. The compound is well-documented for potent anti-inflammatory and antioxidant properties, primarily through pathways like NF-κB and MAPK (43, 44), which are also heavily involved in the host response to *Salmonella* infection (45, 46). Therefore, the observed protection in our mouse model is likely a composite effect, resulting from both the direct inhibition of bacterial virulence *via* InvC and the modulation of the inflammatory response of the host. While our *in vitro* data, which demonstrate high specificity for invasion without affecting bacterial viability or adhesion, strongly support direct anti-virulence as a primary mechanism, dissecting the precise contribution of each component *in vivo* is a critical next step.

However, it is crucial to recognize that anti-virulence agents like SA-B do not possess bactericidal activity; instead, they rely on the host's immune system to clear the disarmed pathogen (47, 48). This dependency makes them potentially unsuitable as a monotherapy for immunocompromised individuals (49, 50). Therefore, investigating the synergistic efficacy of SA-B in combination with traditional antibiotics represents a vital next step, potentially offering a more comprehensive treatment strategy for vulnerable patient populations.

## Experimental procedures

### Molecular docking, virtual screening, and molecular dynamics (MD) simulations

Molecular docking, virtual screening, and MD simulations were performed using the Maestro software suite (Schrödinger LLC). The crystal structure of InvC-ATPγS (PDB ID: 6SDX) was retrieved from the RCSB Protein Data Bank and prepared through a series of steps, including hydrogen addition, protonation state assignment, hydrogen bond optimization, and energy minimization. Ligands, sourced from Chengdu Ruifensi Biotechnology Co., Ltd, were downloaded from PubChem and processed using LigPrep to generate 3D conformations, optimize ionization states (pH 7.0 ± 0.5), and perform energy minimization. Docking studies targeting the ATPγS binding pocket of InvC were conducted using Glide software, employing a tiered approach based on GlideScore ranking: HTVS, followed by SP docking for the top 10%, and XP docking for the best candidates.

MD simulations were performed using Desmond with the OPLS4 force field. Systems were prepared by solvating in a TIP5P water box under periodic boundary conditions, adding counter-ions and 150 mM NaCl to mimic physiological conditions, followed by energy minimization and equilibration. Production simulations were conducted in the NPT ensemble at 300.0 K, maintained by a Nose-Hoover chain thermostat, and 1.01325 bar, controlled by a Martyna-Tobias-Klein barostat with isotropic coupling. A 2.0 fs time step was used for bonded interactions with the RESPA integrator. Short-range non-bonded interactions employed a 9.0 Å cutoff, and long-range electrostatics were calculated using the PME method. Simulations were run for varying durations depending on the specific system studied, as indicated in the results section.

Binding free energies (Δ*G* bind) were calculated on the energy-minimized docked complexes using the Prime MM-GBSA module. To validate the contribution of key residues identified from the MD simulation, an *in silico* K165A mutation was introduced into the wild-type InvC protein structure, and its binding free energy was calculated for comparison. These calculations employed the OPLS4 force field and the VSGB solvation model, retaining the ligand's input partial charges. The total binding free energy was decomposed into its primary energetic components and on a per-residue basis to identify key interaction hotspots.

### SA-B source and preparation

SA-B was obtained from Chengdu Herbpurify Co, Ltd (CAS No. 327-97-9). For preparation of the stock solution, SA-B was solubilized in sterile water to a concentration of 40 mg/ml. For short-term use, SA-B solutions were stored at 4 °C, while for long-term storage, aliquots were frozen at −20 °C.

### Bacterial strains

The bacterial strains utilized in this study encompassed *Salmonella enterica* serovar Typhimurium strain SL1344, *S. flexneri* (*S. flexneri*) serotype 5 strain M90 T, and *E. coli* (*E. coli*) strain PCN033.

### Western blot

Samples were sonicated, separated by SDS-PAGE, and analyzed *via* Western blot. The primary antibodies employed for detection included Flag (1:3000, ABclonal, AE005), FliC (1:1000, Abcam, ab93713), SipA (1:500, prepared in-house), HilA (1:3000, prepared in-house), PrgK (1:1000, prepared in-house), and ICDH (1:3000, Sigma, ABS2090). For secondary detection, AlexaFluor-680 goat anti-mouse IgG H&L (Abcam, ab175775) and AlexaFluor-790 goat anti-rabbit IgG H&L (Abcam, ab175781) were utilized. Blots were visualized using an Odyssey CLx imaging system (LI-COR Biosciences).

### Determination of minimum inhibitory concentration (MIC)

The *S.* Typhimurium culture optical density at 600 nm was set to 0.1, prior to a 100-fold dilution in fresh Luria-Bertani (LB). A 11-point, 2-fold dilution series of SA-B from 0 to 1024 μg/ml was generated in phosphate-buffered saline (PBS), then transferred into a 96-well plate. Following this, 100 μl of the diluted *S.* Typhimurium culture was distributed into each well. Subsequent to incubation at 37 °C for 16 to 20 h, the lowest concentration that inhibited bacterial growth was deemed the MIC for *S.* Typhimurium.

### Plotting of *in vitro* growth curve

*S.* Typhimurium was cultured in LB broth at 37 °C with constant agitation at 220 rpm. Overnight cultures were diluted to an initial optical density at 600 nm (OD_600_) of 0.05 in fresh LB, followed by the addition of SA-B at concentrations ranging from 0 to 128 μg/ml. The OD_600_ readings were recorded hourly over 8 h to plot the growth curve.

### Bacterial invasion, adhesion, and replication assays

HeLa cells were used for all assays. For the invasion assay, *S.* Typhimurium was cultured overnight in LB broth at 37 °C, diluted 1:100 in LB with 0.3 M NaCl and SA-B (0–64 μg/ml), and incubated for 4 h at 37 °C with shaking. As described in earlier studies (39), HeLa cells were infected at a multiplicity of infection (MOI) of 100 for 1 h. The cells were subsequently washed with PBS and treated with Dulbecco's Modified Eagle Medium (DMEM) containing 100 μg/ml gentamicin for 30 min to eliminate extracellular bacteria. Intracellular bacteria were then released with 0.02% saponin and quantified by colony-forming units (CFU) on LB agar plates. The invasion assay for *E. coli* PCN033 was conducted under the same conditions as for *S. Typhimurium*, while an MOI of 50 was used for *S. flexneri* M90T.

For the adhesion assay, the procedure was identical to the invasion assay up to the infection step. Following infection, non-adherent bacteria were removed by washing the cells three times with PBS. The cells were then lysed using 0.02% saponin to release the total bacterial population, which comprised both adherent and intracellular bacteria. The number of adherent bacteria was calculated by subtracting the intracellular bacteria count obtained from the invasion assay from this total bacterial count.

For the intracellular replication assay, HeLa cells were infected at an MOI of 20 for 1 h. After removing extracellular bacteria with 100 μg/ml gentamicin, the medium was replaced with DMEM containing SA-B (0–64 μg/ml) and 20 μg/ml gentamicin. After 24 h of incubation, cells were lysed, and intracellular bacterial replication was quantified by CFU enumeration.

### Immunofluorescence microscopy

HeLa cells were seeded on glass coverslips in 24-well plates and infected with a GFP-expressing *S.* Typhimurium strain as described in the invasion and replication assays. Following infection, cells were washed three times with PBS and fixed with 4% paraformaldehyde for 30 min at room temperature. After fixation, cells were permeabilized with 0.2% Triton X-100 in PBS for 5 min and then blocked with 4% goat serum for 1 h. To visualize the actin cytoskeleton and nuclei, cells were stained with iFluor 594 Phalloidin (Yeasen, 40774ES03) and DAPI, respectively. The coverslips were then mounted on glass slides. Images were captured using an FV3000 confocal microscope. A 100× oil-immersion objective was used for the invasion assay (Fig. 4*E*), and a 40 × objective was used for the replication assay (Fig. 4*F*). Z-stack images were processed and exported using Imaris software (version 10.1.0).

### Cytotoxicity assay

Cytotoxicity was assessed using the Cell Counting Kit-8 (CCK-8; Beyotime, C0038) according to the manufacturer's protocol. Cells were seeded at a density of 5 × 10^4^ cells/well in 96-well plates and incubated overnight. After treatment, cells were incubated with 10 μl of CCK-8 solution for 1 h. The absorbance at 450 nm was measured using a microplate reader. Positive controls included cells treated with 1% Triton X-100 to complete cell lysis. All tests were performed in triplicate, with untreated cells as negative controls.

### Trichloroacetic acid precipitation

*Salmonella typhimurium* cultures were grown overnight at 37 °C, diluted 1:100 in LB with 0.3 M NaCl, supplemented with SA-B to final concentrations of 0, 4, 8, 16, and 32 μg/ml, and incubated for 4 h. Cultures were standardized to an OD_600_ of 1.5. The bacterial suspensions were centrifuged at 14,000*g* for 30 min, and supernatants were collected. Proteins were precipitated by adding TCA to a final concentration of 10% (v/v) and incubating the mixture at 4 °C for 4 h. The precipitated proteins were then collected by centrifugation at 14,000*g* for 30 min. The resulting pellet was washed with acetone and resuspended in 1×SDS loading buffer. The proteins were subsequently analyzed *via* SDS-PAGE to identify differences in protein secretion profiles associated with different treatments.

### Protein purification

Recombinant BL21 (DE3) strains expressing His_6_-tagged InvC or InvC-MUT were grown at 37 °C until OD_600_ reached 0.6 to 0.8. Protein expression was then induced by adding 1 mM isopropyl-β-D-thiogalactopyranoside (IPTG), followed by incubation overnight at 16 °C. Cells were harvested by centrifugation at 5000*g* for 20 min and lysed in a buffer containing 50 mM Tris-HCl (pH 8.0), 150 mM NaCl, and 10 mM imidazole using sonication. The lysate was centrifuged at 12,000*g* for 30 min to separate the soluble proteins. The supernatant was applied to a Ni-NTA column. After washing with 20 mM imidazole (pH 8.0), the His-tagged proteins were eluted using a buffer with 250 mM imidazole (pH 8.0). The protein concentration was determined using the Bradford assay (BIO-RAD, 5000201), and the purity of eluted proteins was analyzed by SDS-PAGE.

### Assessment of InvC enzymatic activity

The enzymatic activity of InvC was assessed by measuring residual ATP levels using the Enhanced ATP Detection Kit (Beyotime, S0027). To determine the IC_50_ and characterize the mode of inhibition, the assay was performed at varying ATP concentrations (0.01, 0.1, and 1 μM). The reaction mixture, in a total volume of 45 μl, contained 50 mM Tris-HCl (pH 8.0), 30 mM KCl, 10 mM ammonium acetate, 1 mM DTT, 5 mM MgCl2, 0.5 mg/ml BSA, 1 μM purified InvC, and SA-B at concentrations ranging from 0 to 256 μg/ml. The mixture was pre-incubated at room temperature for 10 min. The reaction was initiated by the addition of ATP and allowed to proceed at room temperature for 30 min. The reaction was terminated by the addition of the detection reagent, and residual ATP levels were measured following the manufacturer's instructions.

### Thermal shift assay

The effect of SA-B on the thermal stability of InvC was assessed by analyzing protein aggregation. Purified InvC protein (5 μg) was incubated with or without SA-B (32 μg/ml) in PBS (pH 7.4). Samples were heated at temperatures ranging from 25 to 65 °C in 5 °C increments for 5 min. Following heat treatment, samples were centrifuged at 12,000*g* for 30 min to separate the soluble fraction (supernatant) from the aggregated, insoluble fraction (pellet). Both fractions were then collected and analyzed *via* SDS-PAGE with Coomassie Brilliant Blue staining.

### Isothermal titration calorimetry

The binding interactions of the ligands SA-B and ATP with wild-type and mutant (MUT) InvC proteins were analyzed using a MicroCal ITC200 system at 25 °C with a stirring speed of 750 rpm. All proteins and ligands were dissolved in PBS (pH 7.4). For the binding assays, SA-B (1 mM) or ATP (1 mM) was titrated from the syringe into the protein solution (100 μM) in the cell. Titrations consisted of 20 injections of 2 μl with 180-s intervals. The resulting data were fitted using one set of sites binding model within MicroCal Analysis Software to determine the dissociation constant (*K*_D_), enthalpy (Δ*H*), and entropy (Δ*S*).

### *G. mellonella* and mouse infection models

All animal studies were reviewed and approved by the Institutional Animal Care and Use Committee of Jilin University (permit number: SY202412059).

For the *G. mellonella* infection model, groups of 10 larvae weighing 200 to 300 mg were used. For survival studies, larvae were injected with 10 μl of PBS containing 1 × 10^5^ CFU of *S. typhimurium*, while for bacterial load studies, the dose was 5 × 10^4^ CFU. SA-B was administered at doses of 20, 40, or 60 mg/kg starting 30 min post-infection and then every 12 h. Survival was monitored for 60 h.

For the mouse enteritis model, groups of 10 C57BL/6 mice were pre-treated for 3 days with streptomycin administered at 5 g/L in their drinking water. SA-B was administered orally at doses of 20, 40, or 60 mg/kg 2 h before oral challenge with *S. typhimurium*. The challenge dose for survival studies was 1 × 10^7^ CFU, and for bacterial load studies, it was 5 × 10^6^ CFU. Post-infection, SA-B was administered every 12 h for six doses, and survival was recorded daily for 8 days. For bacterial colonization and histopathology, mice were euthanized, and tissues, including the liver, intestines, and spleen, were collected for CFU plating and H&E staining.

### Statistical analysis

Statistical data are expressed as mean ± standard deviation (SD) from at least three independent experiments. Intergroup comparisons were performed using Student's *t* test for pairwise analyses or one-way ANOVA with Tukey's HSD *post hoc* testing for multiple groups. Murine survival data were analyzed using the log-rank test (GraphPad Software). Statistical significance was set at ∗*p* < 0.05; ∗∗*p* < 0.01; ∗∗∗*p* < 0.001; ∗∗∗∗*p* < 0.0001; ns, not significant.

## Ethics statement

The animal study was reviewed and approved by the Institutional Animal Care and Use Committee of Jilin University (permit number: SY202412059).

## Data availability

All the data generated during this study are available upon request from the corresponding authors.

## Supporting information

This article contains supporting information.

## Conflict of interest

The authors declare that they have no conflicts of interest with the contents of this article.
