# Supplementary material for: Structure-based identification of salvianolic acid B as an inhibitor targeting Salmonella InvC ATPase
Source: J Biol Chem. 2025 Sep 15;301(10):110722. doi: 10.1016/j.jbc.2025.110722 (PMC12538069; doi:10.1016/j.jbc.2025.110722)
Supplement: Supporting Information [file mmc2.docx]

**Supporting Information**

**Structure-Based Identification of Salvianolic Acid B as an Inhibitor Targeting *Salmonella* InvC ATPase**

Jiayang Liu, Xinyou Zhang, Kaiyao Zhang, Jianfeng Wang, Xuming Deng, Hongtao Liu, Yanhong Deng, Jiazhang Qiu





**Figure S1. Ramachandran plot analysis of the InvC-ATPγS complex structure, related to Figure 1.** Ramachandran plot for the InvC-ATPγS complex (PDB ID: 6SDX), generated using MolProbity to validate the stereochemical quality of the crystal structure. The plot displays the distribution of backbone dihedral angles (φ and ψ). Dark blue contours indicate favored regions and light blue contours indicate allowed regions.


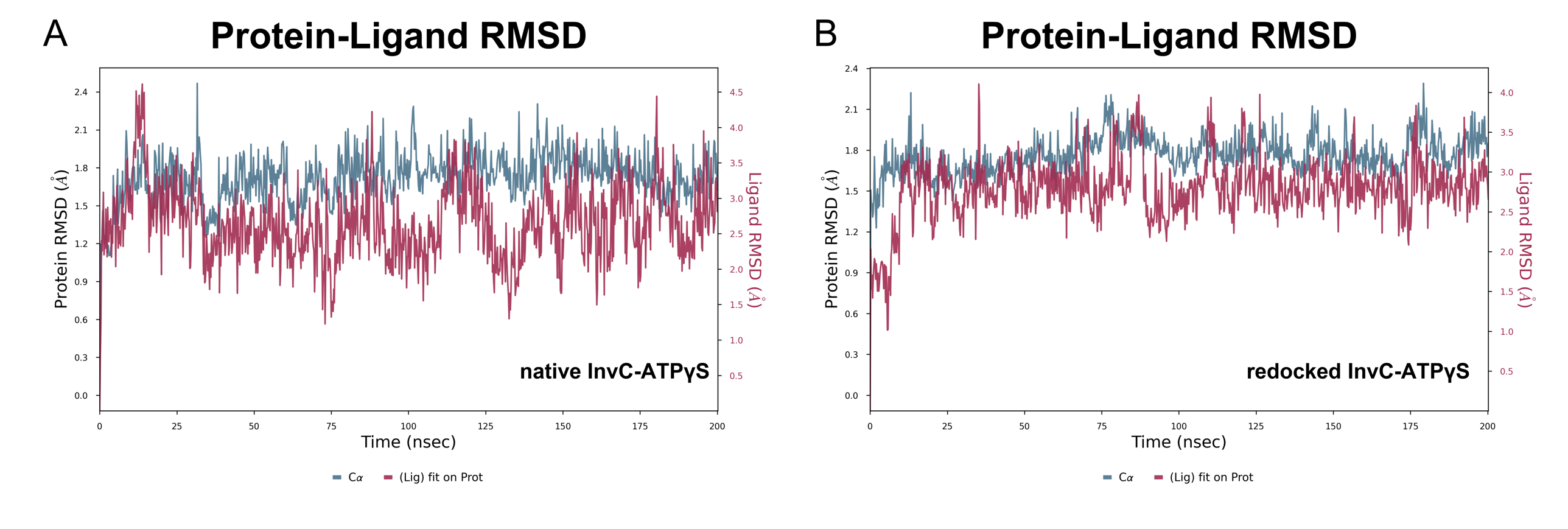


**Figure S2. RMSD analysis of the native and redocked InvC-ATPγS complexes, related to Figure 1.** RMSD plots of the InvC protein backbone (Cα) and the ATPγS ligand over 200 ns MD simulations. These simulations were performed to validate the docking protocol. **A** RMSD profile for the native complex from the crystal structure. **B** RMSD profile for the redocked complex generated by the Glide XP protocol.


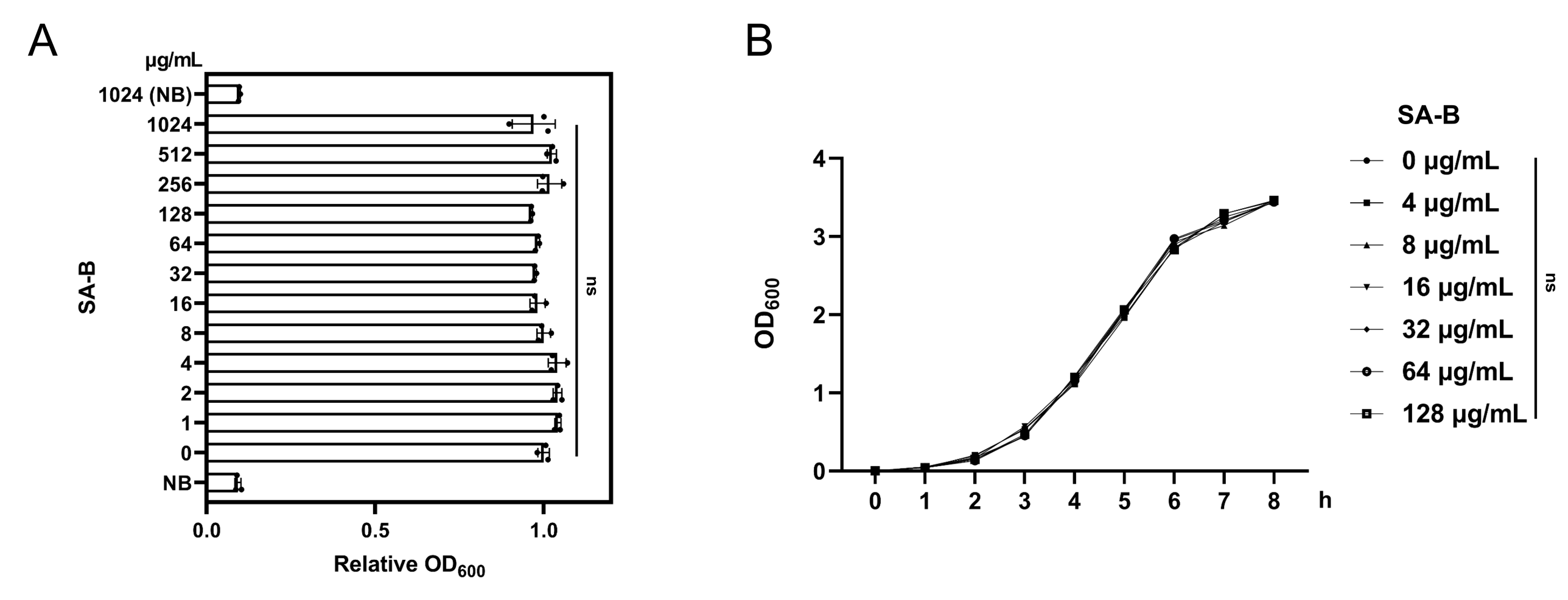


**Figure S3. Effect of SA-B on the growth of S. Typhimurium, related to Figure 4.** **A** MIC assay. *S.* Typhimurium was incubated for 16 h with SA-B at the indicated concentrations, and the relative OD_600_ was measured. **B** Growth curves of *S.* Typhimurium. Bacteria were cultured for 8 h in the presence of SA-B at the indicated concentrations, and the OD_600_ was recorded hourly. Data in **A** and **B** are presented as mean ± SD from three independent experiments. Statistical significance was determined using a one-way ANOVA. NB, no bacteria; ns, not significant.





**Figure S4. Cytotoxicity of SA-B on HeLa cells, related to Figure 4.** HeLa cells were incubated with SA-B at the indicated concentrations. Cell viability was then assessed using a CCK-8 assay. Data are presented as mean ± SD from three independent experiments. A one-way ANOVA was performed. ns, not significant.

**Supporting Information S1**

A list of the 2609 compounds used in the initial virtual screening is provided as a separate Excel file.
